# Uptake of protective tetanus toxoid vaccine doses and maternal associated factors during pregnancy in armed conflict zone, hospital-based cross-sectional study

**DOI:** 10.3389/frph.2024.1353699

**Published:** 2024-07-19

**Authors:** Fassikaw Kebede Bizuneh, Semira Muhidin Mustofa

**Affiliations:** School of Public Health, College of Health Science, Woldia University, Woldia, North East Ethiopia

**Keywords:** armed conflict, Ethiopia, protective dose, pregnancy, tetanus toxoid vaccine

## Abstract

**Background:**

Vaccinating pregnant women with tetanus toxoid (TT) is crucial to prevent neonatal tetanus, reducing related deaths by 94%. In conflict zones with restricted access to deliveries, neonates face a fatality rate of 80%–100%. This study explores the uptake of protective TT vaccine doses and maternal associated factors during pregnancy in an armed conflict zone.

**Methods:**

A hospital-based, descriptive, cross-sectional study was conducted of 357 pregnant women at delivery using simple random sampling. Data were collected through interviews with a structured questionnaire, and entered using Epi-data version 3.1, and exported using SPSS version 22 for further analysis. Binary and multivariable logistic regression analyses were used to identify significant variables for receiving protective TT doses during pregnancy at *P* < 0.05.

**Result:**

In this study, 355 pregnant women were included, with response rate of 99.4%. The mean age of the participants was 27.65 ± 6.23 years. During the study period, 67.3% of pregnant women received a protective TT vaccine dose while 33.3% were missed due to escalated armed conflict. The dropout rates were significant from TT5 to TT2 (17.6%), TT5 to TT3 (11.9%), and TT5 to TT4 (6.1%). However, maternal associated factors for the uptake of the TT protective vaccine dose were identified, including being aged 36–49 years [adjusted odds ratio (AOR) = 3.7; 95% confidence interval (CI) 1.54–7.8; *P* = 0.001], completing high school (AOR = 3.05; 95% CI 1.5–8.9; *P* = 0.02), having an antenatal care follow-up (AOR = 9.4; 95% CI 2.9–24.3; *P* = 0.001), previous media exposure (AOR = 15.5; 95% CI 7.5–25.3; *P* = 0.001), and good maternal knowledge (AOR = 2.7; 95% CI 1.8–4.9; *P* = 0.02).

**Conclusion:**

The uptake of the protective TT vaccine dose among pregnant women in a continued armed conflict area was low compared with previous study findings. Efforts should be made to increase vaccine uptake and reduce dropout rates by addressing both community and individual-level factors.

## Introduction

Tetanus is caused by a neurotoxin produced by the bacterium *Clostridium tetani* and has a fatality rate of 80%–100% (1, 2). Immunizing pregnant women or women of childbearing age with two doses of the tetanus toxoid (TT) vaccination can potentially reduce neonatal tetanus mortality by 94% (3, 4). To prevent tetanus-related deaths, pregnant women should receive a minimum of three doses of tetanus-specific immunoglobulin (TIG) (5, 6). The World Health Organization (WHO) recommends five TT vaccine doses for individuals with unknown immunization status (7). In the first pregnancy, two doses of the TT vaccine are recommended, with the first dose given during the first antenatal care (ANC) visit and the second dose administered 4 weeks later (8–10). This immunization provides anti-tetanus antibodies for both mother and infant antibodies, transferred across the placenta as IgG, offers protection, and with 0.1–0.15 IU/ml is advised (1, 2, 11).

Globally, tetanus is a significant public health issue, especially in areas with low immunization coverage and limited access to clean deliveries. Each year, an estimated 3.3 million neonatal deaths occur worldwide, with 34,019 deaths attributed to tetanus, as reported by the WHO in 2017 (4, 12). Each year, approximately 303,000 women die from pregnancy-related complications, leading to approximately 830 deaths per day, as per the latest estimates from the WHO (13, 14). The majority (90%) of maternal and neonatal morbidity of tetanus occurred in countries in Southeast Asia and sub-Saharan Africa (15, 16). In India alone, 14,751 cases of tetanus were reported in 2019 (17).

A systematic review and meta-analysis conducted in Ethiopia revealed that only 52.6% of pregnant women received at least two doses of the TT vaccine (6, 13) with significant regional disparities, including 72.5% in SNNPR (18), 39.2% in Oromia (14), 56.2% in Debre Tabor and Amhara regions (19), and 40.2% in Tigray (20). Previously, tetanus infections accounted for 24% of neonatal mortality, a range of 14–31 per 1,000 live births (1, 3, 10). Previous research conducted in Ethiopia (1, 3, 8, 9, 21) has identified factors associated with deterring the protective dose of the TT vaccine, including late initiation of ANC, no ANC visit, poor wealth index, lack of media exposure, maternal unemployment, rural residence, and high community illiteracy (3). A study on reproductive-aged women indicated the dropout rate for TT was reported at 55.6% (TT1 to TT5). The dropout rates were 5.3% for TT1–TT2, 14.7% for TT2–TT3, 20.2% for TT3–TT4, and the highest, 31.1%, for TT4–TT5 (4, 22).

In Ethiopia during the civil war of North East Ethiopia (between July 2021 and 28 November 2023), the Amhara region was heavily impacted, with 50% of healthcare facilities damaged, resulting in interrupted maternal health services for 70,000 pregnant women (11, 22) and including 1.4 million displacements and 600,000 reported death (1, 22). The civil war impacted delivery of the TT vaccination service in areas of conflict due to security issues and the destruction of healthcare facilities (7, 10). Therefore, the aim of this hospital-based, cross-sectional study was to assess the level of uptake of the protective TT vaccine dose and maternal factors during pregnancy in an armed conflict zone of Saint Lalibela.

### Methodology

#### Study area

A facility-based, cross-sectional study was conducted at St. Lalibela General Hospital between 16 February and 7 March 2023. Situated in Lalibela town, the hospital serves the capital of the Lasta Lalibela district in the North Wollo Zone of northern Ethiopia. It is located 622.5 km away from Addis Ababa and 310 km from Bahir Dare. The hospital provides healthcare services to a population of approximately 600,000 people annually. The study area was selected due to the long-lasting armed conflicts during the civil war, which resulted in numerous disruptions, including over 1,000 recorded civilian deaths and injuries in the region.

#### Study design

A hospital-based, cross-sectional study was employed among 357 pregnant women in St. Lalibela General Hospital.

#### Inclusion criteria

All pregnant women receiving delivery services at St. Lalibela General Hospital during the study period were included in the interviews. However, mothers who were not pregnant and seeking maternal and child health services, including family planning or care for illnesses, at the hospital were excluded from the study.

#### Operational definition

Uptake of the protective TT vaccine dose refers to the protection provided to pregnant women who have received at least two doses of TT (2, 23); or tetanus toxoid protective dose immunization (TTPDI) is defined as the proportion of mothers who have received any of the following documented TT doses: (1) two TT injections during the pregnancy; (2) two or more injections, with the last one administered within 3 years of the birth; (3) three or more injections, with the last one administered within 5 years of the birth; (4) four or more injections, with the last one administered within 10 years of the birth; or (5) five or more injections at any time before the birth (2).

#### Neonatal tetanus

Neonatal tetanus typically occurs within 3–21 days of birth and is caused by the introduction of tetanus spores through the umbilical cord during delivery, often as a result of using unclean materials to cut the cord (24).

#### Mass media exposure

Reproductive-aged women were asked about their exposure to TT vaccine messages through television, radio, newspaper, and mobile messages. To simplify the analysis, a variable called “exposure to mass media messages” was created, coded as “yes” if respondents had previous exposure to these messages (25).

#### Dependent variables

The dependent variable in this study is TT vaccine uptake (Yes/No).

#### Independent variables

The independent variables included sociodemographic factors (age, marital status, level of education, occupation, residence, and income), obstetric and related factors (nulliparity, planned pregnancy, pregnancy-related complications), health and health-related factors (distance from the health facility, ANC follow-up), and media exposure.

#### Sample size calculation

The sample size of this study was estimated using a single population proportion formula: *n* = (z^2^ × *p* × (1 − *p*))/*d*^2^. Based on a prevalence of 69.8% reported from Gondar University Hospital in 2019 (10), with a 95% confidence interval (CI; *z* = 1.96) and a margin of error of 5% (*d* = 0.05), the formula yielded a sample size of approximately 324. Finally, after considering a non-response rate of 10%, the final sample size was 357.

#### Methods of data collection technique

Pregnant mothers who received immediate postnatal care were interviewed, excluding severely deaf and mentally ill individuals. The questionnaire was prepared in English and translated into Amharic. Face-to-face interviews were conducted using the Kobo Toolbox, involving data collectors, supervisors, and investigators. The interview questions covered sociodemographic data, conflict-related factors, health facility-related information, and TT vaccination. A pre-test was conducted at Woldia Health Centre with 18 mothers (5% of the sample size) to ensure questionnaire consistency. The questionnaire was translated back to English from Amharic to ensure understandability and consistency. Data collection was carried out by a public health supervisor and two trained nurses. The interview questions focused on sociodemographic data, such as age, marital status, educational status, occupation, economic status, residence, number of children, and ANC follow-up, vaccination information, distance from the health facility, media exposure related to vaccination, and history of previous ANC follow-up and immunization.

#### Data processing and analysis

Data were checked for completeness and consistency before analysis. The Kobo toolbox was used for data collection, and the collected data were exported to SPSS version 25 for further analysis. The descriptive analysis determined means, frequency, and percentage distributions. Bivariate and multivariable logistic regressions were conducted. Independent variables with a *p*-value < 0.25 in bivariate logistic regression were included in the multivariable logistic regression, considering collinearity and data normality using a stepwise backward elimination procedure. The model's fitness was assessed using the Hosmer–Lemeshow goodness-of-fit test. Categorical variables with adjusted odds ratios (AOR) and 95% CIs were selected as determinants of TT vaccine coverage at *p* < 0.05. Knowledge about the TT vaccination was assessed using nine structured questions, and a mean score was calculated. Scores below the mean were considered poor knowledge, while scores above the mean were considered good knowledge. The Cronbach alpha test was conducted, and the overall knowledge-related assessment questions yielded a *p*-value of 0.76 (12, 26, 27).

## Results

### Sociodemographic characteristics

Out of 357 postnatal women, 355 were interviewed, resulting in a response rate of 99.5%. The participants had a mean age of 27.65 ± 6.23 years, with the majority (72.4%) in the age range of 26–34. Of the participants, 91 (37.3%) had completed primary education or higher. Over half of the participants (91.5%) were married, with 220 (62.2%) being housewives. The majority of respondents (89.1%) identified as Orthodox religious followers, while 110 (30.9%) had to travel over 10 km to reach a health institution for ANC care (Table 1).

**Table 1:** Socio-demographic characteristics of study participants in St. Lalibela General Hospital for protective doses of TT vaccine.

Variables	Category	Frequency	Percent (%)
Maternal age	15–25	37	10.4
	26–35	257	72.4
	36–49	61	17.2
Educational status	Unable to read and write	34	9.6
	Can read and write	39	11.0
	Complete high school	91	25.6
	Preparatory and above	191	37.7
Occupation status	Housewife	220	62.1
	Civil servant	67	18.8
	Merchant	61	15.8
	Others	7	2.0
Religion	Orthodox	316	89.1
	Muslim	29	8.2
	Protestant	10	2.8
Marital status	Married	325	91.5
	Single	16	4.5
	Widowed	8	2.3
	Divorced	6	1.7
Family income	≤5,000 birr per month	281	79.3
	≥5,000 birr per month	74	20.5
Family numbers	≤3 members	1,157	44.7
	>4 members	198	55.3
Residence	Urban	202	56.9
	Rural	153	43.1
Distance from HC	<1 h	245	69.7
	≥1 h	110	30.9
Media exposure	Yes	187	52.3
	No	168	57.7
Maternal parties	First birth	57	16.2
	Multi-parities	298	83.5
ANC status	No ANC attendant	59	16.6
	ANC 1st	128	36.1
	ANC 2nd–3rd	98	27.6
	ANC 4th completed	70	19.7
Pregnancy status	Planned	267	75.2
	Unplanned	88	24.8

### Maternal and health service-related factors

Of the total participants, majorities 267 (71.6%) of women had response for planned pregnancies and received support from their families. Moreover, 298 (83.5%) postnatal women were multiparous women, 70 (33.8%) completed fourth ANC visit, but 59 (16.6%) did not have any ANC follow-up.

### Knowledge about the TT vaccine

The majority of participants (84.5%) were aware of the benefits of the TT vaccine, with 80.6% knowing that it is free of cost. In addition, 52.3% of participants had previous exposure to media messages about the importance of TT vaccine uptake. More than half (64.5%) believed that the TT vaccine had dual benefits for infants. Furthermore, 81.7% were aware that tetanus can be caused by unsafe delivery and injuries. Lastly, 73.5% of participants had good knowledge about the recommended TT vaccination dose for the protection of neonates and mothers (Table 2).

**Table 2:** Level of maternal knowledge about TT vaccine protective doses for participant s women in St. Lalibela General Hospital for.

Variables		Categories	Frequency	Percent
1	Have you ever heard about the TT vaccine?	Yes	301	84.8
		No	54	15.2
2	Do you know TT vaccines are free of cost given in the health institution	Yes	286	80.6
		No	69	19.5
3	Do you know TT Vaccine is important for infant and mother	Yes	230	64.5
		No	125	35.2
4	Are you aware that tetanus disease causes death in both mother and newborn	Yes	298	83.9
		I don’t know	60	16.9
5	Do you know tetanus is serious and can transmitted during unsafe delivery	Yes	287	81.6
		No	68	18.5
6	Do you know tetanus can be transmitted from soil, dust, and manure injuries	No	82	23.1
		Yes	273	76.9
7	Did you hear TT five dose can be only prevented lifelong from tetanus	Yes	192	54.8
		No	163	45.5
8	Do tetanus toxoid (TT) vaccine can prevent TT disease	Yes	256	72.1
		No	99	27.8
9	To whom priorities would be given TT vaccine	Females & children	267	75.2
		Males at any age	88	24.8
10	Respondent’s TT vaccine knowledge scores	Good	261	73.5
		Poor	94	26.5

### Impact of armed conflict on protective TT dose and dropout

In this study, more than half of the participants 258 (57.5%) experienced food insecurity, and 16.3% (59) lost at least one family member during the escalated war of Tigray people liberation front (TPLF) with Northern parts of Amhara region (Table 3).

**Table 3:** Burden of maternal individual characteristics factors during escalated war among participant women in St. Lalibela General Hospital, 2023.

	Characteristics	Categories	Frequency	Percent
1	Household food security	Secured	187	52.68
		Insecure	168	47.32
2	Lost family members due to the war	Yes	59	16.6
		No	296	83.5
3	Family displaced due to war	Yes	270	76.1
		No	85	23.9
4	Did you start ANC before the war	Yes	296	83.3
		No	59	16.6
5	If you started ANC, did you get TT	Yes	195	66.6
		No	101	34.1
6	TT vaccination status	Over all TT given for pregnant	239	67.3
		Completed over all TT5 uptake	23	6.4
		Over all dropped out (TT2–TT4)	216	60.8
		Ever TT vaccine missed women	116	32.7

TT1, tetanus toxoid 1 (Given to all females through community campaign) and TT2, tetanus toxoid 2 (started for pregnant women at health institution by considering TT1 received from the community and schools levels).

**Figure 1:**
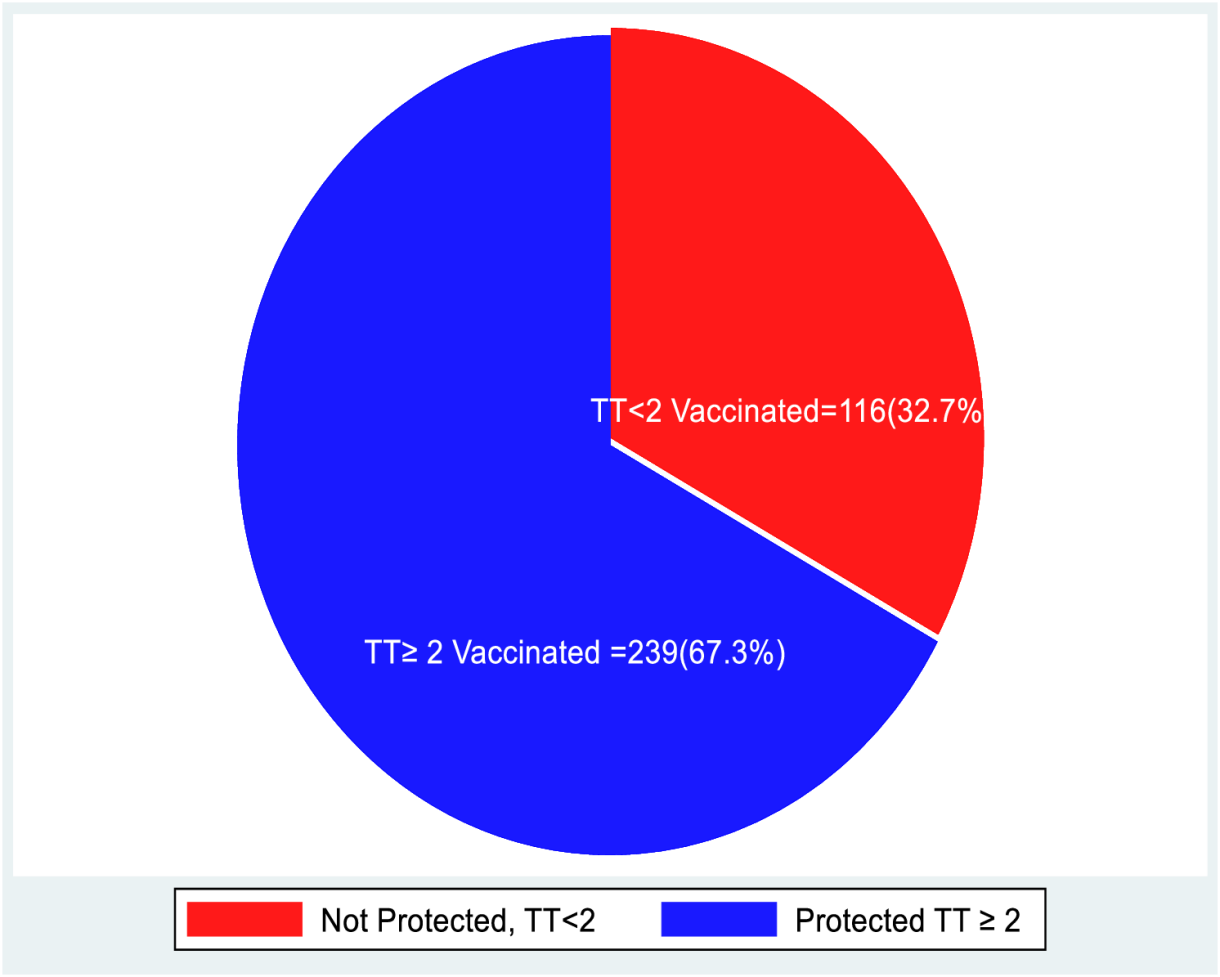
TT vaccine protective dose uptake by postnatal women in St. Lalibela General Hospital.

The overall TT vaccine protective dose uptake was found 67.3% received the protective doses (TT2), nonetheless only 6.4% participant's women completed TT5 dose (Figure 1).

**Figure 2:**
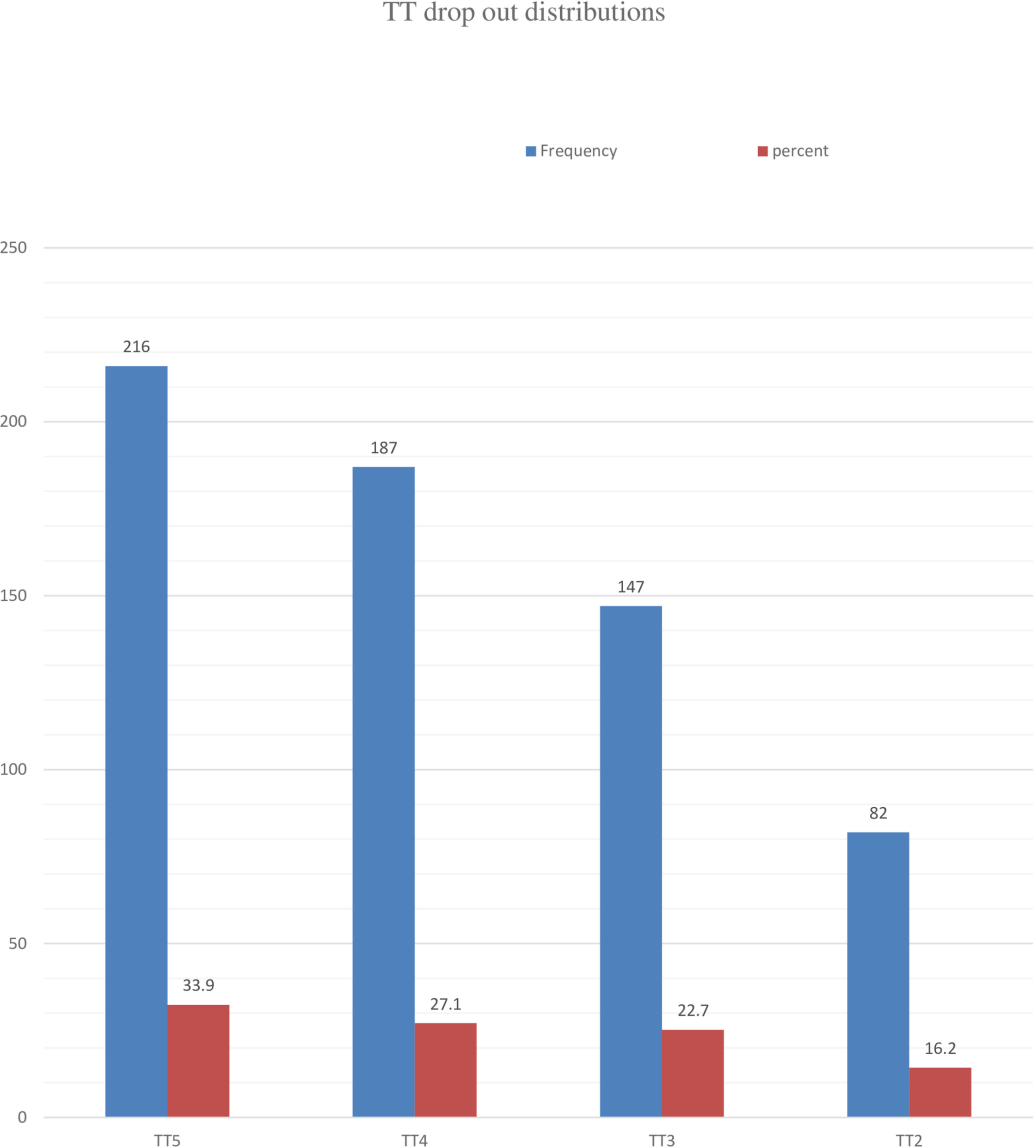
Distribution of dropped TT doses among postnatal women who received care at St. Lalibela.

During the interview, significant dropout rates were observed across different TT uptake intervals, with 17.6% from TT5 to TT2, 11.9% from TT5 to TT3, and 6.1% from TT5 to TT4, attributed to reasons such as war outbreaks (41.6%), forgetting their schedule (31.4%), and fear of side effects (28.2%), indicating notable variation in TT vaccine uptake interval (Figure 2).

### Maternal associated factors for uptake of the protective TT vaccine dose

In the regression analysis, variables associated with the coverage of a protective dose of TT vaccine were identified using a significance threshold of *p* < 0.25. Eight variables, including age, residence, pregnancy status, level of education, occupation, number of ANC visits, and marital status, were included in the final multivariable logistic regression model. The results showed that age, knowledge of the TT vaccine, previous mass media exposure, level of education, and fourth ANC follow-up status were significantly associated with achieving protective TT doses within a 95% CI. Specifically, mothers aged 15–25 years were 3.7 times more likely to receive the protective dose compared to those aged 36–49 years (AOR = 3.7; 95% CI 1.54–7.8; *p* = 0.001). Completing high school or above (AOR = 3.05; 95% CI 1.5–8.9; *p* = 0.02), having the fourth ANC follow-up (AOR = 9.4; 95% CI 2.9–24.3; *p* = 0.001), exposure to media for TT (AOR = 15.5; 95% CI 7.5–25.3; *p* = 0.001), and having good knowledge (AOR = 2.7; 95% CI 1.8–4.9; *p* = 0.02) were also significantly associated with achieving the protective dose of TT2 during pregnancy (Table 4).

**Table 4:** Factors associated with uptake of protective tetanus toxoid vaccine doses among mothers during pregnancy in St. Lalibela Hospital, North Wollo Zone, Northeast Ethiopia.

Variables	Categories	TT vaccine status		COR (95%CI)	AOR (95%CI)	*P*-value
		Not protected	Protective (≥TT2)			
Age	15–25	11	26	3.58 (1.74–6.87)	3.7 (1.54–7.8)	0.001*
	26–35	74	183	2.42 (1.41–4.43)		
	36–49	31	30	Ref		
Resident	Rural	63	139	Ref		
	Urban	53	100	1.32 (0.73–1.78)		
Educational status	Read and write	63	77	Ref		
	Highschool & above	53	192	2.57 (1.55–3.83)	3.05 (1.5–8.9)	0.02*
Occupation	Civil servant	92	133	Ref		
	Farmer/others	24	106	3.15 (1.85–5.21)		
House hold food security	Secured	56	131	1.28 (0.856–2.07)		
	Insecure	60	108	Ref		
ANC follow up	No F/up	33	26	Ref		
	1	55	72	1.68 (0.91–3.1)		
	2–3	20	78	4.96 (2.43–10.1)	1.6 (0.9–11.6)	0.05
	4th	8	60	8.23 (3.61–20.49)	9.4 (2.9–24.3)	0.001*
Lost f/member on war	Yes	54	5	Ref		
	No	62	234	2.79 (1.54–6.95)		
Displaced during war	Yes	84	53	Ref		
	No	32	186	1.21 (0.51–1.39)		
Pregnancy	Planned	76	189	1.95 (1.19–3.198)		
	Unplanned	40	50	Ref		
Parity status	Nulliparous	59	99	Ref		
	Multiparous	57	140	1.45 (0.92–2.23)		
Media exposure on TT	Yes	30	202	16.5 (9.35–27.7)	15.5 (7.5–25.3)	0.001*
	No	86	36	Ref		
Knowledge of TT	Poor	23	71	Ref		
	Good	93	168	3.11 (1.92–7.06)	2.7(1.8–4.9)	0.021*

* significant variables during regression.

## Discussion

Tetanus is a vaccine-preventable disease that can occur in all populations, with neonates and pregnant women at highest risk. Ethiopia has the highest maternal and neonatal tetanus morbidity and mortality rates and only 49% of mothers get vaccinated with adequate tetanus toxoid in Ethiopia (3). Despite growing recognition of the detrimental effects of conflict on maternal and child healthcare, the literature is bereft of studies robustly quantifying the association between conflict and TT protective dose uptake of pregnant women during ANC service in Ethiopia, particularly in the Amhara region (8, 9). The continued civil war in Ethiopia, specifically in the Amhara region, has led to extensive damage to healthcare facilities, disrupting maternal health services for more than 70,000 pregnant women, causing 1.4 million displacements and 600,000 reported deaths (11, 22).

The conflict affected the delivery service of TT vaccination services due to security concerns, disrupting resource allocations, and facility destruction. This study estimated the level of TT vaccine uptake and maternal associated factors during the escalated war in St. Lalibela Hospital.

At the end of the study period, 67.3% (95% CI 62.3–72.0) of pregnant women received a protective dose of the TT vaccine. This report is in line with the previous study findings in Gondar Hospital (69.8%) (10), Debre Birhan Town (72.3%) (28), Damboya, SNNR (72.5%) (18), in Sudan (73.7%) (1), and North India (68%) (29). However, this finding is higher than previously reported in Debre Tabor (56.2%) (30), Tigray (40.2%) (20), Oromia (39.2%) (14) and Somali (51.8%) (6), and EDHS (42.4%) (9). The observed discrepancy in TT vaccine coverage rates between this study may be attributed to time variations and differences in maternal knowledge.

On the other hand, the final report of this study is lower than previously reported in the Lao People's Democratic Republic (79.7%) (31) and in Nigeria (81.1%) (32). The variation in TT vaccine coverage rates could be attributed to cultural differences, health-seeking behavior, healthcare coverage, economic and infrastructural access, and the impact of war. In our study, out of 296 pregnant women who initiated ANC before the war outbreak, 239 (67.1%) received a protective dose of the TT vaccine, while only 23 (6.4%) completed the full TT vaccination. In addition, 270 (76.3%) pregnant women were temporarily displaced from their residences during the war (16).

On the other hand, there was a significant TT dropout recorded at each TT uptake interval; this revealed the overall dropout rates from TT5 to TT2 (TT5–TT2), from TT5 to TT3 (TT5–TT3), and from TT5 to TT4 (TT5–TT4) were 17.6%, 11.9%, and 6.1%, respectively, with significant variation among each consecutive interval of TT vaccine uptake for pregnant women. This is consistent with the findings in previous studies conducted among pregnant women with TT vaccine uptake in Sudan (21) and Sierra Leone (33). The possible elucidation for this significant difference between each consecutive interval of TT vaccine uptake was related to the continued armed conflict in both Ethiopia and Sudan, which led to larger coverage of TT for pregnant women.

Maternal factors associated with achieving a protective dose of the TT vaccine during pregnancy were identified; accordingly, pregnant women aged 15–25 years had a three times higher likelihood of receiving the protective dose compared to those aged 36–49 years. This is consistent with previous findings in the southern region (SNNR) (8) and in Sudan (1). The possible reasons for the increased likelihood of younger pregnant women, particularly those aged 15–25 years, to be more likely to receive the TT vaccine during ANC is due to their curiosity, attentiveness, and proactive approach toward their pregnancy and them actively seeking out the vaccine as they believe it offers crucial protection during pregnancy.

In this study, pregnant women who completed high school have a 3.05 times higher likelihood of receiving the protective dose compared to illiterate pregnant women. This report is consistent with previous findings in Ethiopia (13), Tigray, Ethiopia (20), Debre Tabor town (30), and a Somali region (6). When increased level of education the more likely hood of aware women about to importantly of TT vaccine protective doses for infant and increased the likelihood of intention to took it during pregnancy.

The findings of the study revealed that mothers who attended their fourth ANC visits were 9.4 times more likely to receive two doses of TT injection than mothers who attended fewer than two ANC visits. This is consistent with the findings of previous studies in southern Ethiopia (8), Amhara region, and Tigray region (14, 20). This might be due to the pregnant mothers having regular ANC visits, the likelihood of receiving comprehensive healthcare services and completing the recommended TT vaccine doses, and increased contact time with healthcare professionals who provide mothers with valuable health information, which can positively influence their healthcare-seeking behavior.

There was a significant association between pregnant women and previous media exposure. This study found that mothers who were exposed to media about the importance of the TT vaccine were more likely to receive the protective dose (AOR = 15.5; 95% CI 7.5–25.3; *p* = 0.001). This is consistent with a systematic review conducted in Ethiopia (13) and in previous national-level studies (3, 9). This might be because media exposure increases awareness, knowledge, and accurate information about the benefits of TT vaccination during pregnancy. It helps address misconceptions and cultural beliefs, improves access to healthcare services, and builds trust between healthcare providers and pregnant women.

Consistent with previous findings in Egypt (34), Gondar (2), a Somali region (35), and Sierra Leone (33), pregnant women with a good knowledge of protective TT vaccination doses have a 2.7 times higher likelihood of increased uptake of protective doses compared with the pregnant group of pregnant women (AOR = 2.7; 95% CI 1.8–4.9; *p* = 0.02). This might be the pregnant mothers had regular ANC visits, the probability of getting comprehensive and completed the recommended healthcare service including, the probability of getting full TT vaccine protective doses had increased. Healthcare providers, public health campaigns, and educational initiatives play a crucial role in disseminating accurate information about the vaccine's benefits and safety.

Unlike the findings in previous studies (1, 8, 9, 21), in this study, family income, displacement during war, and the number of families did not significantly affect the uptake of protective TT vaccine doses for pregnant women. However, the pregnant women's knowledge of the tetanus vaccine and their level of education significantly impacted the uptake of the vaccine, highlighting the importance of addressing these factors to improve immunization rates, reduce hesitancy, and promote a culture that values vaccination.

The present study has some limitations. The cross-sectional survey design makes it difficult to establish clear causal relationships between the factors described and uptake of the TT vaccine. Recall bias may have influenced the study's results, as participants did not present their vaccination cards during data collection. Nevertheless, the study offers valuable insights to inform the development of health education interventions targeting increased uptake of the TT vaccine among pregnant women in Ethiopia.

## Conclusion and recommendation

The utilization of the protective TT vaccine dose among mothers in the study area was low. Factors that enhanced the protective TT dose included level of education, ANC visits, age of respondent, fourth ANC follow-up, media exposure, and good knowledge about TT. Low utilization of the protective TT vaccine dose among pregnant in the study area can be addressed by focusing on raising awareness about regular ANC visits, establishing mobile clinics or outreach programs for healthcare access, enhancing security through collaboration, conducting community-based education campaigns involving community and religious leaders, providing training on safe vaccine administration, fostering coordination among healthcare providers and organizations, supporting healthcare facilities, and recognizing the psychological impact of conflict and offering mental health support services.

## Data Availability

The original contributions presented in the study will be made available upon reasonable request. Further inquiries can be directed to the corresponding author.
